# Gesture-Controlled Image Management for Operating Room: A Randomized Crossover Study to Compare Interaction Using Gestures, Mouse, and Third Person Relaying

**DOI:** 10.1371/journal.pone.0153596

**Published:** 2016-04-15

**Authors:** Rolf Wipfli, Victor Dubois-Ferrière, Sylvain Budry, Pierre Hoffmeyer, Christian Lovis

**Affiliations:** 1 Division of Medical Information Sciences, Geneva University Hospitals, Geneva, Switzerland; 2 Division of Orthopaedics and Trauma Surgery, Geneva University Hospitals, Geneva, Switzerland; 3 University of Geneva, Faculty of Medicine, Geneva, Switzerland; University of Groningen, University Medical Center Groningen, NETHERLANDS

## Abstract

**Objective:**

In this work, we aim at comparing formally three different interaction modes for image manipulation that are usable in a surgery setting: 1) A gesture-controlled approach using Kinect ®; 2) oral instructions to a third part dedicated to manipulate the images; and 3) direct manipulation using a mouse.

**Materials and Methods:**

Each participant used the radiology image viewer Weasis with the three interaction modes. In a crossover randomized controlled trial participants were attributed block wise to six experimental groups. For each group, the order for testing the three modes was randomly assigned. Nine standardized scenarios were used.

**Results:**

30 physicians and senior medical students participated in the experiment. Efficiency, measured as time used to pass the scenario, was best when using the mouse (M = 109.10s, SD = 25.96), followed by gesture-controlled (M = 214.97s, SD = 46.29) and oral instructions (M = 246.33s, SD = 76.50). Satisfaction, measured by a questionnaire, was rated highest in the condition mouse (M = 6.63, SD = 0.56), followed by gesture-controlled (M = 5.77, SD = 0.93) and oral instructions (M = 4.40, SD = 1.71). Differences in efficiency and satisfaction rating were significant. No significant difference in effectiveness, measured with error rates, was found.

**Discussion:**

The study shows with formal evaluation that the use of gestures is advantageous over instructions to a third person. In particular, the use of gestures is more efficient than verbalizing instructions. The given gestures could be learned easily and reliability of the tested gesture-control system is good.

**Conclusion:**

Under the premise that mouse cannot be used directly during surgery, gesture-controlled approaches demonstrate to be superior to oral instructions for image manipulation.

## Background and Significance

Operating rooms (OR) are filled with electronic equipment providing the intervention team control over instruments and information on patients’ health condition. Those devices are operated by surgeons, nurses, anesthesiologists, and technical assistants. A special requirement for these non-sterile devices is that they are only used by team members who are scrubbed. Accordingly, surgeons cannot directly manipulate the most common user interfaces, such as a mouse and a keyboard, during intervention without specific sterile packaging. These devices are known to be highly contaminated [1]. Thus, surgeons will often rely on workarounds to perform tasks such as manipulating images. A solution is to scrub out when interacting with a computer. However, this is time consuming and prolongs surgery time. It is also possible to put sterile covers over the mouse and the keyboard, but this considerably decreases the usability. Further, surgeons can use the inside of their gown to contact the keyboard, keeping the outside of their clothes sterile. This procedure can be risky and is not satisfying [2]. Finally, and the most often used solution is to rely on somebody who is present and executes required manipulations, guided by the surgeon. However, explaining the exact position of an area of interest and communicating the best viewing parameters is often difficult and time consuming [3].

Fortunately, there are alternatives to the classic mouse/keyboard interaction mode. Natural interfaces, which can interpret human action without direct contact, are on the rise. One of the obvious means of natural communication is voice control. They are used in OR, but problems are reported due to the noisy operating environment [4]. A controlled experiment was conducted to evaluate the effectiveness of an OR environment control system, comparing voice control interface, touch panel control, and giving order to a person. Voice control was significantly slower than touch panel control and relaying to a third person. In contrast to this objective finding, participants subjectively evaluated voice control as quickest. Finally, voice control suffers low reliability when non-native speakers use the recognition engine [3].

Gesture-controlled manipulation is another way of interacting with images, and it has received a lot of attention recently with the availability of low-cost devices used in the gaming landscape. Johnson et al. [2] evaluated the potential for gesture-controlled interactions in interventional radiology, illustrated the problems raised when guiding an assistant verbally, and proposed requirements for such systems. A gesture-controlled system has to discriminate the communication gestures between people in the OR and the ones for image control; it needs to capture movements of the hand relative to the body because physicians need to move freely around the table; and finally it should accommodate input from different team members. First tests with gesture control devoted for the OR were made in 2004 [3]. The system used stereo cameras to detect motion in a given three-dimensional work space. A mock-up interface was developed to test the interactions with the gesture-based menu control. Sixteen participants of varied backgrounds executed non-medical scenarios. It was shown that users quickly learned to use gestures to navigate and a menu point selection took less than 5 seconds. Whereas in first studies, gestures were used to move a mouse pointer, a more powerful interaction form consists of relating a specific gesture with a command. Studies showed the feasibility of such systems [5]. Wachs et al. [6] conducted an in vivo experiment for a biopsy planning task. The methods for evaluation included contextual interviews, individual interviews, and subjective satisfaction questionnaires. The surgeons were overall satisfied. In a second experiment with students, the same authors report a recognition accuracy of 96% and the efficiency of 22 seconds per task after 10 trials.

Starting 2010, the availability of the Kinect® device, developed by Microsoft for gamers, eased further research on gestures-controlled interactions. Kinect® is an inexpensive off-the-shelf motion tracker. The device can capture video data in 3D under any ambient light conditions using an infrared laser depth sensor combined with a monochrome CMOS sensor. It also features a multi-array microphone so that it can provide full-body 3D motion capture, facial recognition and voice recognition capabilities. Gallo et al. [7] describe how the sensor functionality can be used to control imaging data with body movements. Recently, finger movements could be tracked [8].

One of the challenges raised in gestures-control is the reproduction of switch actions, such as “click” or “enter”. Ruppert and colleagues [9] developed two prototypes for interaction with imaging data. In a first prototype, they used the hold still to click paradigm and, in a second, they used one hand for interactions and the other hand to activate virtual clicks.

There are already a few reports about Kinect ® in the OR. Strickland et al. [10] tested it with surgeons during laparoscopic and open surgery procedures. According to the surgeons, the most useful functionality was the ability to intraoperatively animate CT and MRI scans and switch to different series within a study. They also reported that zooming, rotating, highlighting points of interest, and annotating are less useful. During the interventions it was observed that IR radiation of overhead lights may interfere with the capture qualities of Kinect ®. Ebert et al. [11] evaluated a gesture recognition system for CT imaging visualization. They report that it took 1.4 times longer to complete the task with the Kinect® than with the mouse. Usability satisfaction with the gestures control was rated 3.4 out of 5. The rating 5 was given by the authors for the interaction with the mouse being a gold standard and communicated it so to participants. Tan et al. [12] conducted a usability study with 29 radiologists. They used a two hand manipulation paradigm. 5 specific tasks for abdominal CT inspection were solved. 69% of the participants found the solution useful and a 20% found it easy to use and another 58% reported that they found it moderately difficult.

User-defined gestures are generally easier to remember than pre-defined gestures [13]. However, there is a legacy bias when users define the gestures themselves: they choose the gestures that resemble what they are used to so far, for example mouse movements [14]. Jacob et al. [15] initiated their research with an ethnographic study including 10 surgeons. In a first step, surgeons suggested 21 gestures. In a second step, they agreed on 10 gestures out of the 21. The system took into account contextual information such as position of thorax and head, and position of arms. After training, a mean gesture recognition accuracy of 94% could be achieved. Bigdelou and colleagues [16] studied user-defined gestures using Kinect® with 12 participants from a general population. The test required to locate an aortic stent in CT and navigate to its bifurcation. They evaluated the pragmatic and hedonic quality of the system. However, the authors could not show that personalized gestures could significantly improve pragmatic and hedonic quality.

In research by Hötker et al. [17], ten radiological residents interacted with 10 CT guided punctures and 18 angiography using Kinect® with voice or gestures-control commands. The gesture control was evaluated with a score 7.7/10 for satisfaction and 6 out of 10 physicians would use it every day.

## Objectives

It has been shown that -gesture-based interaction is technically feasible and that user satisfaction was generally good. The objective of this work is to compare experimentally the use of gestures-control of imaging data with the two common alternative methods: relaying to a third person and direct input with a mouse, the latter being the gold standard. This work applies the methodologies previously published by Ebert et al. [11] and Punt et al. [4] for comparing different interaction modes for systems destined for use in the OR.

Usability is defined as the “extent to which a product can be used by specified users to achieve specified goals with effectiveness, efficiency and satisfaction in a specified context of use” [18]. In our trial, we will measure effectiveness by the achievement of a goal with as few errors as possible, efficiency by the time to reach the goal, and satisfaction with a questionnaire.

There are thus three conditions: a) gesture control; b) a third person executing the orders given by a surgeon and c) using a non-sterile mouse, the gold standard. In the text, these three groups will be referred to as “*Gesture”*, *“Mouse”* and *“Third”*.

The study was approved by the human research ethics commission of the Geneva University Hospitals (HUG).

## Materials and Methods

### System details and system in use

At the Geneva University Hospitals (HUG), two systems are used routinely for image manipulation, namely OsiriX on the Mac platform and Weasis on all platforms. Weasis is a multiplatform Java-based system with basic imaging capabilities. Osirix and Weasis are both open source projects that have been initiated at HUG.

Currently, manipulation of medical imaging during surgery is done by a third person under the surgeon’s instructions. Alternatively, the surgeon has to scrub out and do himself the manipulations.

KiOP (Kinect in the OPerating room) is non-commercial software developed at HUG that enables Weasis to be manipulated with Microsoft Kinect®. The system was developed based on surgeons’ needs by a team composed of two surgeons, two radiologists and three IT engineers.

The system is composed of three components:

1. Motion-sensing device (Kinect®, Microsoft);
2. DICOM viewer (Weasis, Open Source: https://www.openhub.net/p/weasis);
3. KiOP software.

A screenshot of the system (Weasis + KiOP) can be found in S1 Fig. The Kinect® sensing device has been chosen because it has sufficient precision and response time, is low-cost, and is well-documented, and provides a programming interface.

The integration between the motion sensor and the DICOM viewer is achieved by messages transmitted by KiOP from the device to the viewer after interpretation. The messages of the device are extended to include zoom, window and level, pan, series scroll, layout and mouse control. The messages use the telnet network protocol, thus achieving maximum interoperability. The DICOM viewer receives the messages and converts them to actions.

For the purpose of this test we choose depersonalized X-rays and CT images used in the division of traumatology. They were chosen by VDF in order to have a sample of typical images.

### Study Design

The study design was a crossover randomized controlled trial (RCT) with blocked randomization. By picking a paper slip, participants are allocated to one of the 6 experimental groups, each with a size of 5 participants.

Each block has a different order of interaction modes. Interaction modes are direct use of Weasis with the mouse (condition *Mouse*), use of Weasis by giving verbal instructions to a third person (condition *Third*), or using Weasis with the gestures-control system KiOP (condition *Gesture*).

The choice of a RCT is guided by the main research question, which is assessing and comparing efficiency, effectiveness, and user satisfaction of the three different interaction modes. We used a crossover design in order to let the participants compare the interaction modes in the subjective evaluation part of the study.

### Measurement

For evaluation of user satisfaction the *After-Scenario Questionnaire* (ASQ) by Lewis was chosen [19]. ASQ score is a brief 3-item questionnaire that measures using a 7-step Likert response format (1 = strongly disagree, 7 = strongly agree) the components ease of task completion, time to complete a task, and adequacy of support information.

The quantitative approach was accompanied by a qualitative approach which is not reported here.

### Participants

Physicians and medical students were recruited in the Division of Orthopedics and Trauma at HUG and in the Faculty of medicine at the University of Geneva. The inclusion criterion was that they have used Weasis more than three times in the past. The exclusion criteria was participants’ prior use of the gesture-based tool KiOP. Participation is voluntary without financial incentives.

### Power Analysis

As the main outcome, the variable efficiency was used. A power analysis showed that we need a sample of 26 participants to show a difference. This calculation was made with a beta of 0.2 and an alpha of 0.05. Effect size was estimated using two distributions from a prior study [20] where two prototypes of a clinical information system were tested. We used means of those distributions and the geometric mean of corresponding standard deviations to calculate the required sample size. In order to have equal numbers of participants in each group, a total of 30 participants was targeted.

### Study Flow

**Fig 1:**
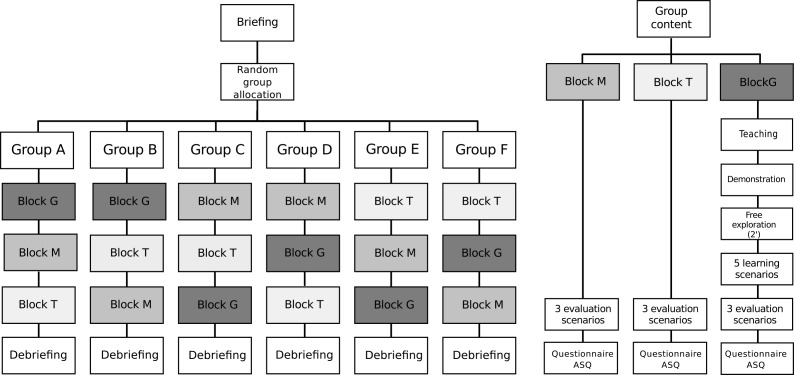
Study flow. Interaction modes: G = gesture, M = mouse, T = third.

The study took place between 28 Feb 2014 and 22 May 2014. Physicians and medical students were asked to visit the usability laboratory (Evalab) at HUG. Participants were first introduced to the procedure and the goal of the study. After formal acceptance, they were attributed randomly to one of the experimental groups by drawing a slip (Fig 1).

The slip was not put back so each block was filled with 5 participants. The 9 test scenarios were randomized using the random function in Microsoft Excel. The test scenarios were randomized across participants. Hence, in each experimental group, there was the same order of interaction modes but with a different order of test scenarios (Fig 1).

For each of the three interaction modes, *Mouse* and *Third*, and *Gesture*, there were three test scenarios compared to each other. With the interaction mode *Gesture* there was an additional teaching phase, a free learning phase, and 5 learning scenarios before the participants started with the test scenarios.

The teaching was scripted and consisted of a presentation of slides that explained the use of KiOP and a demonstration by the experimenter. Subsequently, the participants could freely explore the functionalities of KiOP for 2 minutes in the free learning phase. In both the teaching and the free learning phase, the participant could ask questions on the use of KiOP to manipulate images. In the following learning and test phase questions were allowed. In the test phase, participants’ time was limited to 30 seconds per task.

The order of the learning scenarios was kept constant, starting with a first simple scenario that required three tasks and then continuing with more complex scenarios (see scenarios in S1 Table).

### Methods for data acquisition and measurement

The time spent on each interaction mode was measured using Techsmith Morae™. During the experiment, time markers are set at the beginning and end of each interaction mode. At the end of data acquisition, the Morae software reports the time spent per interaction mode.

Effectiveness is the measure of the correctness of executing a scenario. The experimenter rated the performance. There was a maximum number of points per scenario, which were the number of tasks per scenario (see scenarios in S1 Table). The activation and deactivation of KiOP was not counted as a task in order to keep the same number of total tasks per scenario. Each task correctly executed is counted as one point. In the following cases the point was not counted: choosing the inappropriate tool, failing to execute the task within 30 seconds, or losing track by Kinect. The maximum score per interaction mode could change between 3 and 12 for learning scenarios, and between 7 and 9 for test scenarios. Therefore, effectiveness was calculated as the percentage of correct tasks (100 * correct/max points. Finally, we calculated the average percentage score for each interaction mode which could range from 0 to 100.

The user satisfaction was measured with an adapted version of the After Scenario Questionnaire (ASQ) [19]. The slightly modified questions were:

1. “Overall, I am satisfied with the ease of completing the tasks in this scenario.”
2. “Overall, I am satisfied with the amount of time it took to complete the tasks in this scenario.”
3. “Overall, I am satisfied with the way the system guided me to complete the tasks in this scenario.”

The third question was modified because there is no support information included in the system as the original ASQ implies. Also, this question was not asked after the interaction mode *Third* as there was no guidance by the system. Finally, the modified questionnaire was translated into French. The ASQ is measured on a Likert scale from 1 [strongly disagree] to 7 [strongly agree].

Following variables were gathered using a questionnaire given to the participants at the beginning of the introduction: age, sex, job title, months of experience with Weasis, and last use of Weasis.

### Outcome measures

Our main outcome was efficiency. It was operationalized as the time spent in seconds solving the scenarios in each interaction mode. Further, we evaluated efficacy, operationalized by subtraction of points when errors were committed in solving the scenarios and finally the user’s satisfaction reported by participants in each interaction mode.

### Methods for Data Analysis

For all intra-subject comparisons, a Shapiro-Wilk test was applied in order to evaluate whether the distributions are normal. In the ASQ score, data was treated as an interval scale because the distances between the 7 steps of the response were equal [21].

Each analysis was paired between *Gesture* and *Mouse*, *Gesture* and *Third*, and *Mouse* and *Third*. For distributions that did not meet the criteria for parametric tests, the corresponding non-parametric tests were used.

Regarding the efficiency, all distributions were normal and a paired two-sided t-test was applied. In order to determine significant differences, we used a Bonferroni corrected alpha = 0.017.

The efficacy, measured by scores, showed a strong ceiling effect and the distribution was not normal. Therefore, the Wilcoxon signed rank test was used to compare the means.

All differences in ASQ scores between conditions were normally distributed, except: [ease] *Gesture*–*Mouse* and [system guidance] *Gesture*–*Mouse*. For the normal distributions, a paired two-sided t-test was applied. The question on system guidance was unclear to the participants and only descriptive statistics were applied.

## Results

Thirty participants were recruited and participated in the experience; 18 were men and 12 were women. There were 13 advanced medical students (years 5 and 6), 14 residents, 1 attending physician, and 2 deputy heads of division. Participants had used the DICOM viewer Weasis in average for 17.9 months (SD = 20.38) Professional experience ranged from 0 years for medical students to 20 years for one of the deputy head of division.

Efficiency, meaning a short time passed on the test scenarios, was highest (M = 109.10s, SD = 25.96) when using *Mouse*, followed by *Gesture* control (M = 214.97s, SD = 46.29) and *Third* (M = 246.33s, SD = 76.50). A two-sided paired t-test showed that the differences between all pairs were significant (p = 0.014 for the difference between *Gesture*–*Third* and p < 0.001 for *Mouse*–*Gesture* and *Mouse*–*Third*.

The mean relative test score revealed that the highest score was in the condition *Mouse* (M = 98.31, SD = 4.541) followed by *Third* (M = 96.81, SD = 5.81) and *Gesture* (M = 96.45, SD = 4.54), without reaching significance in a Wilcoxon test. There was a strong ceiling effect of scores (see Table 1) and no further analysis on learning performance was conducted.

**Table 1:** Effectiveness evaluated as a percentage of successful tasks with learning and test scenarios.

	Learning scenarios					Test scenarios								
Condition	*Gesture*					*Gesture*			*Mouse*			*Third*		
Scenario	1	2	3	4	5	1	2	3	1	2	3	1	2	3
M	84.44	95.71	93.70	91.67	96.11	97.67	95.18	96.51	98.57	98.31	98.04	96.51	96.72	97.20
SD	20.96	7.64	8.60	10.26	6.83	5.59	9.08	7.03	4.36	4.43	4.48	8.66	8.42	5.98

Usability satisfaction was evaluated with questions about ease of use, efficiency, and system guidance on a scale from 1 [strongly disagree] to 7 [strongly agree]. Ease of use was rated highest in the condition *Mouse* (M = 6.63, SD = 0.56), followed by *Gesture* M = 5.77, SD = 0.93), and *Third* (M = 4.40, SD = 1.71). Paired Wilcoxon tests showed significant differences in the following pairs: *Mouse-Gesture*, *Gesture*-*Third*, and *Mouse*-*Third* (all pairs p < 0.001).

Regarding efficiency, participants subjectively rated that they were most efficient with *Mouse* (M = 6.77, SD = 0.43) followed by *Gesture* (M = 5.47, SD = 1.14) and *Third* (M = 4.00, SD = 1.762). The difference between all pairs was significant with a paired two-sided t-test.

Finally, system guidance was evaluated slightly higher in the condition *Mouse* (M = 6.33, SD = 0.92) than *Gesture* (M = 6.13, SD = 1.04), but this difference was not significant with a paired Wilcoxon test. Also, this question was often not understood by participants.

Regarding the demographic variables, no correlation of work experience with satisfaction or task performance could be found.

## Discussion

The study showed that the use of gestures is advantageous over instructions to a third person. In particular, the use of gestures was more efficient than verbalizing instructions. Also, participants were more satisfied with it and made only insignificantly more errors than when relaying to a third person. A control condition was used where participants have been using a mouse without sterile cover. As expected the results showed that participants are more efficient with the mouse than with gestures-control. In our study the participants were 2.2 times faster using the mouse than using KiOP.

Regarding the effectiveness, we could not determine any significant difference between the three conditions. A ceiling effect was present in scores in the learning scenarios. Only few errors were made and the only notable difference was between the first and second learning scenario. Even if the experiment had been constructed in order to measure a learning effect, it failed to do so. The study supports previous studies that showed that pre-defined gestures with Kinect® can be learned easily [12, 17] and that reliability of gesture-control systems is good [6]. However, in a next study difficulty of scenarios should be increased.

Regarding user satisfaction, participants were most satisfied with the ease of use and efficiency with *Mouse*, rather satisfied with *Gesture*, and least satisfied in *Third*. Our results are in line with previous studies with gesture-control systems showing good evaluations in terms of ease of use [12, 16], although lower than using a mouse [11, 17]. The fact that giving order to a person was the least preferred interaction mode was a surprise. We would have expected that it is simpler to explain to somebody than using a new interaction mode, namely KiOP. A positive subjective evaluation of efficiency when using gestures could be shown. This finding is in line with the measured efficiency per scenario. The third question of ASQ on system guidance was not clear to participants, so no interpretation of the result is possible.

The strength of our study is the methodology that enables to test objectively with a significant sample of the target population. A gesture-control system is compared to the current way of using DICOM viewers in OR.

The limitations of our experiment are that it has not been made during surgery, but using a simulated environment. Another limitation is that participants were not blind about the study goals, which could induce a complacency bias.

## Conclusion

Our work compared gesture-control, use of mouse and oral instructions given to a person to use a DICOM viewer in an OR setting. Efficiency, effectiveness, and user satisfaction have been evaluated. The study demonstrated that gesture-control worked better than oral instructions to a person, but not better than mouse usage. We believe that the difficult conditions of using a mouse in a sterile will further promote the use of gesture interfaces. The study can inform decision makers whether to integrate a gesture-control system in an OR using evidences rather than enthusiasm. We hope this work will help improving the work conditions of physicians and, as a consequence, patient safety.

## Supporting Information

S1 Fig Screenshot of user interface (Weasis + KiOP). Tools in the lower part are from left to right: reset image, split window, move, change contrast, zoom, scroll through slices, point, select, and quit.
(TIF)

S1 File Permission to publish S1 Fig. (PDF)

S1 Table Scenarios. (PDF)
