# Supplementary figures and images for: Gesture-Controlled Image Management for Operating Room: A Randomized Crossover Study to Compare Interaction Using Gestures, Mouse, and Third Person Relaying
Source: PLoS One. 2016 Apr 15;11(4):e0153596. doi: 10.1371/journal.pone.0153596 (PMC4833285; doi:10.1371/journal.pone.0153596)

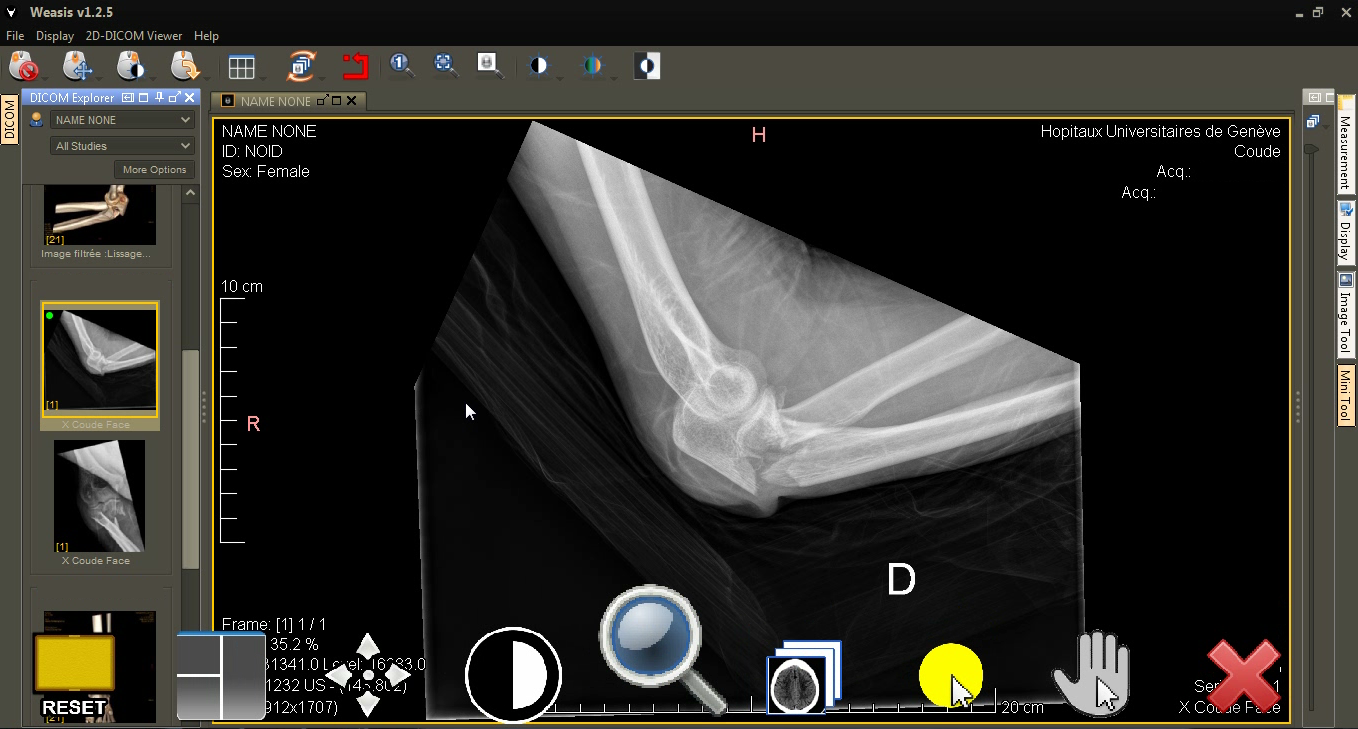

Supplement: S1 Fig — Tools in the lower part are from left to right: reset image, split window, move, change contrast, zoom, scroll through slices, point, select, and quit. (TIF) [file pone.0153596.s001.tif]
