# Supplementary material for: Gesture-Controlled Image Management for Operating Room: A Randomized Crossover Study to Compare Interaction Using Gestures, Mouse, and Third Person Relaying
Source: PLoS One. 2016 Apr 15;11(4):e0153596. doi: 10.1371/journal.pone.0153596 (PMC4833285; doi:10.1371/journal.pone.0153596)
Supplement: S1 File — (PDF) [file pone.0153596.s002.pdf]

Hôpitaux
Universitaires
Genève

Rue Gabrielle-Perret-Gentil 4
1211 Genève 14
Switzerland

Department of
surgery

Division of
Orthopaedics and
Trauma Surgery

Permission to publish

S1 Fig: Screenshot of user interface (Weasis + KiOP) was taken by Victor Dubois-Ferrière and can be reused under CC BY Licence

Victor Dubois-Ferrière
Attending physician
