# Supplementary material for: Gesture-Controlled Image Management for Operating Room: A Randomized Crossover Study to Compare Interaction Using Gestures, Mouse, and Third Person Relaying
Source: PLoS One. 2016 Apr 15;11(4):e0153596. doi: 10.1371/journal.pone.0153596 (PMC4833285; doi:10.1371/journal.pone.0153596)
Supplement: S1 Table — (PDF) [file pone.0153596.s003.pdf]

Learning scenarios

Scenario	Task	Score
**Scenario 1**<br><br>Image used: X-ray of elbow frontal/profile		
Starting point: X-ray frontal view	Open session (hand waving like in all following session openings)	0
	Zoom on elbow:<br>Selection<br>Utilization<br>Quit	 <br>1<br>1<br>1
	Close session	0
Max. score		3
**Scenario 2**<br><br>Image used: X-ray of elbow frontal/profile		
Starting point: X-ray profile	Open session	0
	Zoom in on image:<br>Selection<br>Utilization<br>Quit	 1<br>1<br>1
	Increase contrast:<br>Selection<br>Utilization<br>Quit	 1<br>1<br>1
	Reset view:<br>Utilization	 1
	Close session	0
Max. Score		9
**Scenario 3**<br><br>Image used: X-ray of elbow frontal/profile		
Starting point: X-ray profile view	Open session	0
	Selection X-ray profile:<br>Selection<br>Utilization<br>Quit	 1<br>1<br>1
	Centering of image on radial head:<br>Selection<br>Utilization<br>Quit	 1<br>1<br>1
	Zoom on radial head:<br>Selection<br>Utilization<br>Quit	 1<br>1<br>1
	Close session	0
Max. score		9
**Scenario 4**<br><br>Image used: X-ray of elbow frontal/profile + CT elbow		
Starting point: X-ray profile	Open session	0
	Add sagittal view of CT:<br>Selection<br>Utilization<br>Quit	 1<br>1<br>1
	Scroll to the fracture of radial head:<br>Selection<br>Utilization<br>Quit	 1<br>1<br>1
	Split screen vertically:<br>Selection<br>Utilization<br>Quit	 1<br>1<br>1
	Add image X-ray profile:<br>Selection<br>Utilization<br>Quit	 1<br>1<br>1
	Close session	0
Max. score		12
**Scenario 5**<br><br>Image used: X-ray of elbow frontal/profile + CT elbow		
Starting point: CT 3D	Open session	0
	Rotate CT in 3D (360 deg.):<br>Selection<br>Utilization<br>Quit	 1<br>1<br>1
	Add X-ray profile of elbow<br>Selection<br>Utilization<br>Quit	 1<br>1<br>1
	Zoom on olecranon:<br>Selection<br>Utilization<br>Quit	 1<br>1<br>1

	Point on olecranon:<br>Selection<br>Utilization<br>Quit	 <br>1<br>1<br>1
	Close session	0
Max. score		12

Test scenarios

Scenario	Task	Score
**Scenario 1**<br><br>CT of elbow with coronal, axial slices and 3D		
Starting point: CT sagittal slide	Open session (hand waving like in all following session openings)	0
	Scroll to fracture of ulna<br>Selection<br>Utilization<br>Quit	 <br><br>1<br>1<br>1
	Zoom on fracture:<br>Selection<br>Utilization<br>Quit	 <br>1<br>1<br>1
	Center on olecrane<br>Selection<br>Utilization<br>Quit	 <br>1<br>1<br>1
	Close session	0
Max. score		9
**Scenario 2**<br><br>Image used: CT with coronal, axial slices and 3D		
Starting point: CT with sagittal slice	Open session	0
	Split screen vertically:<br>Selection<br>Utilization<br>Quit	 <br><br>1<br>1<br>1
	Add coronal slices in the right pane:<br>Selection<br>Utilization<br>Quit	 <br><br><br><br>1<br>1<br>1
	Increase contrast:<br>Selection<br>Utilization<br>Quit	 <br>1<br>1<br>1
	Close session	0
Max. Score		9
**Scenario 3**<br><br>Image used: CT of elbow with coronal, axial slices and 3D		
Starting point: CT with sagittal slice	Open session	0
	Selection 3D recon-struction:<br>Selection<br>Utilization<br>Quit	 <br><br>1<br>1<br>1
	Rotate to frontal view of elbow:<br>Selection<br>Utilization<br>Quit	 <br><br><br>1<br>1<br>1
	Zoom on elbow:<br>Selection<br>Utilization<br>Quit	 <br>1<br>1<br>1
	Close session	0
Max. score		7
**Scenario 4**<br><br>Image used: X-ray of knee frontal and profile		
Starting point: X-ray profile	Open session	0
	Point to patella:<br>Selection<br>Utilization<br>Quit	 <br>1<br>1<br>1
	Center on letter D in image:<br>Selection<br>Utilization<br>Quit	 <br><br>1<br>1<br>1
	Open frontal X-ray:<br>Selection<br>Utilization<br>Quit	 <br><br>1<br>1<br>1
	Close session	0
Max. score		9
**Scenario 5**<br><br>Image used: X-ray of knee frontal and profile		
Starting point: X-ray frontal	Open session	0

	Split screen vertically<br>Selection<br>Utilization<br>Quit	 1<br>1<br>1
	Add X-ray profile<br>Selection<br>Utilization<br>Quit	 1<br>1<br>1
	Modify contrast of X-ray profile:<br>Selection<br>Utilization<br>Quit	 1<br>1<br>1
	Close session	0
Max. score		9
**Scenario 6**<br><br>Image used: X-ray of knee frontal and profile		
Starting point: X-ray frontal	Open session	0
	Zoom<br>Selection<br>Utilization<br>Quit	 1<br>1<br>1
	Modify contrast:<br>Selection<br>Utilization<br>Quit	 1<br>1<br>1
	Reset image:<br>Utilization	 1
	Close session	0
Max. score		7
**Scenario 7**<br><br>Image used: X-ray of elbow and CT		
Starting point: X-ray profile	Open session	0
	Point on fracture:<br>Selection<br>Utilization<br>Quit	 1<br>1<br>1
	Center on letter D:<br>Selection<br>Utilization<br>Quit	 1<br>1<br>1
	Add X-ray frontal:<br>Selection<br>Utilization<br>Quit	 1<br>1<br>1
	Close session	0
Max. score		9
**Scenario 8**<br><br>Image used: X-ray elbow and CT		
Starting point: X-ray frontal	Open session	0
	Zoom on elbow<br>Selection<br>Utilization<br>Quit	 1<br>1<br>1
	Decrease contrast, making X-ray more grayish<br>Selection<br>Utilization<br>Quit	 1<br>1<br>1
	Reset image:<br>Utilization	 1
	Close session	0
Max. score		9
**Scenario 9**<br><br>Image used: X-ray profile of elbow and CT 3D		
Starting point: CT 3D	Open session	0
	Rotate image (360 deg.)<br>Selection<br>Utilization<br>Quit	 1<br>1<br>1
	Zoom<br>Selection<br>Utilization<br>Quit	 1<br>1<br>1
	Add X-ray profile:<br>Selection<br>Utilization<br>Quit	 1<br>1<br>1
	Close session	0
Max. score		9
